# Immunomodulatory Effects of Cinnamaldehyde in *Staphylococcus aureus*-Infected Wounds

**DOI:** 10.3390/molecules28031204

**Published:** 2023-01-26

**Authors:** Cristiane Santos Silva e Silva Figueiredo, Patrícia Vieira de Oliveira, Warlison Felipe da Silva Saminez, Roseana Muniz Diniz, Juliana Silva Pereira Mendonça, Lucas dos Santos Silva, Miria Yasmim Miranda Paiva, Mayara de Santana do Nascimento, Amanda Silva dos Santos Aliança, Adrielle Zagmignan, João Francisco Silva Rodrigues, Joicy Cortêz de Sá Souza, Marcos Augusto Grigolin Grisotto, Luís Cláudio Nascimento da Silva

**Affiliations:** 1 Laboratório de Patogenicidade Microbiana, Universidade Ceuma, São Luís 65075-120, Brazil; 2 Rede de Biodiversidade e Biotecnologia da Amazônia Legal (BIONORTE), São Luís 65075-120, Brazil; 3 BD Research Center Ireland Castletroy, Co., V94 V500 Limerick, Ireland

**Keywords:** cinnamon, essential-oil components, skin lesions, bacterial infections, skin wounds

## Abstract

Cinnamaldehyde (CNM) is an essential-oil component with reported anti-infective, anti-inflammatory, and healing effects, making it an interesting compound for the treatment of wound infection. Herein, we evaluated the effects of topical administration of CNM in experimental wounds infected by *Staphylococcus aureus*. *Swiss* mice (*n* = 12/group) were randomly allocated into three groups (CON: animals with uninfected lesions; Sa: animals with untreated infected lesions; Sa + CNM: animals with infected wounds and treated with CNM). Excisional lesions (64 mm^2^) were induced at the dorsal area followed by the addition of *S. aureus* (80 μL of a 1.5 × 10^8^ CFU/mL bacterial suspension). The wounds were treated with CNM (200 μg/wound/day) or vehicle (2% DMSO) for 10 days. Skin samples were taken on the 3^rd^ or 10^th^ treatment day for quantification of inflammatory mediators, bacterial load, immunophenotyping, and histological analysis. The treatment with CNM improved the healing process and attenuated the severity of skin lesions infected by *S. aureus*. These effects were associated with significant decreases in bacterial loads in CNM-treated wounds. The levels of neutrophils, TNF-α, IL-6, NO, and VEGF were decreased in the lesions treated with CNM. Taken together, these data provide further evidence of the effectiveness of CNM for the treatment of skin infections.

## 1. Introduction

Despite significant advances in therapeutic resources for healing, skin wounds still impose great costs on the health systems worldwide [1]. The presence of pathogens, such as *Staphylococcus aureus*, in the injured skin tissue, induces an intense inflammatory response that predisposes individuals to the development of chronic wounds and delays the tissue repair process [2,3,4]. In these situations, there is an intense production of inflammatory mediators (such as TNF-α and nitric oxide (NO)) that promotes the recruitment of leukocytes to the wound site. The excess of leukocytes and inflammatory mediators may promote more damage and impair healing [5,6].

**Figure 1:**
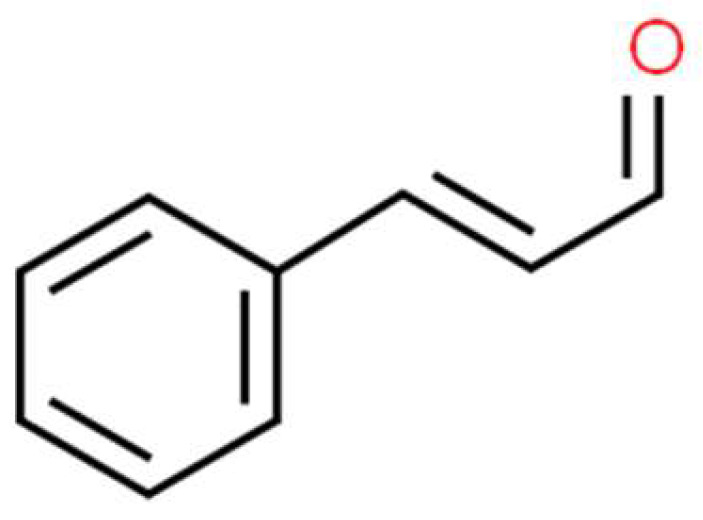
Structural formula of cinnamaldehyde (C_9_H_8_O). The structure was obtained from ChemSpider (ID553117).

In the context of the treatment of contaminated wounds, it is important that the drug candidate has healing and/or immunomodulatory effects, in addition to its antimicrobial action [3,7,8]. Several pieces of evidence suggest that cinnamaldehyde (CNM; Figure 1), the main compound of cinnamon essential oil (obtained from *Cinnamomum* sp.), can fulfill all these requirements [9,10,11,12]. CNM has antimicrobial and antivirulence properties towards *S. aureus*, reducing biofilm viability, adhesion ability, and release of cytotoxic substances [13,14,15].

Despite the antivirulence potential of CNM against *S. aureus*, its effects in vertebrate models of infection caused by this pathogen have not been reported. Previous data show that the topical treatment with CNM accelerated the tissue repair process of wounds contaminated by *Pseudomonas aeruginosa*, an effect associated with a decrease in the severity of the infection and mediated by the interaction with the TRPA-1 receptor (transient receptor potential ankyrin 1) [10]. The arsenal of virulence determinants involved in skin infections induced by *S. aureus* and *P. aeruginosa* are different, as well as the mechanisms of immune response [2,16]. In this sense, the present study aimed to evaluate the effects of topical treatment with CMN on experimental skin wounds contaminated with *S. aureus*.

## 2. Results

### 2.1. Topical Treatment with Cinnamaldehyde Reduces the Healing Time of Wounds Contaminated by S. aureus

**Figure 2:**
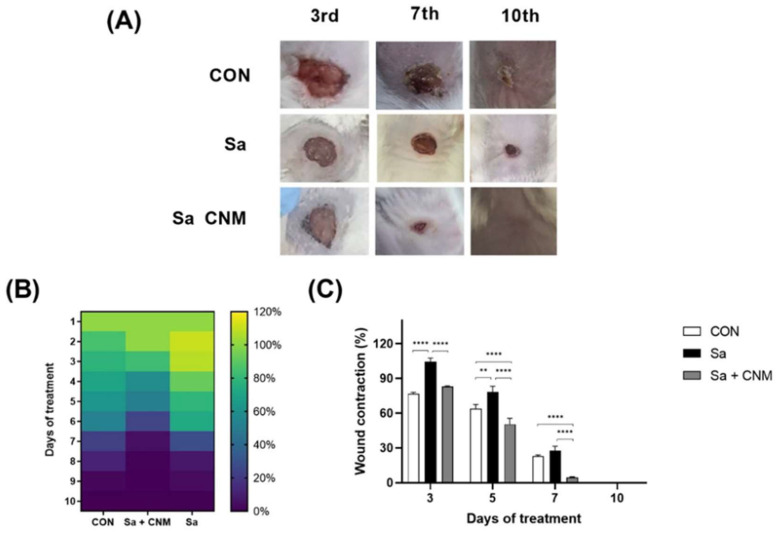
Effects of topical treatment with cinnamaldehyde in skin lesion contaminated by *Staphylococcus aureus.* (**A**) Macroscopic evaluation of the healing process of the experimental groups used in this study. (**B**) Heat map of relative wound contraction during 10 days of treatment; (**C**) Relative wound contraction of selected days. CON: animals with uninfected skin lesions; Sa: animals with skin lesions infected with *S. aureus*; Sa + CNM: animals with skin lesions infected with *S. aureus*. ** *p* < 0.01; **** *p* < 0.0001.

In this study, *Swiss* mice (*n* = 12/group) were randomly allocated into three groups (CON: animals with uninfected lesions; Sa: animals with untreated infected lesions; Sa + CNM: animals with infected wounds and treated with CNM). The evolution of lesion sizes and their macroscopic aspects were analyzed daily during the ten days of treatment (Figure 2A). Initially, it is possible to notice that the wounds of the Sa group presented higher wound size than those in the CON group. This effect was more evident between the 2nd and 6th days when these two groups showed statistical differences (*p* < 0.05; Figure 2B). The topical treatment with CNM significantly improved the healing of wounds contaminated by *S. aureus* (Figure 2B,C). The differences were significant between the 3rd and 7th days (*p* < 0.001).

**Figure 3:**
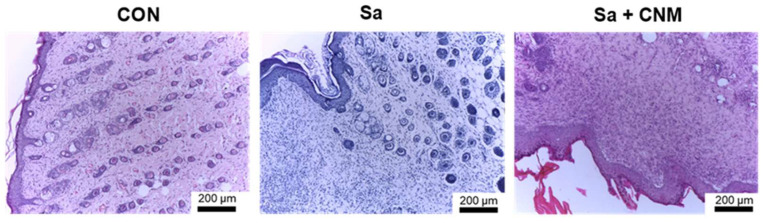
Histological analysis after three days of topical treatment with cinnamaldehyde in skin lesion contaminated by *Staphylococcus aureus.* CON: animals with uninfected skin lesions; Sa: animals with skin lesions infected with *S. aureus*; Sa + CNM: animals with skin lesions infected with *S. aureus*.

Histological analysis showed that the CNM-based therapeutic protocol intensified the healing process, with the re-epithelialization process well-evidenced after 10 days of treatment. At this stage, it was possible to observe that the epidermis presented similar patterns to the control group, that is, it is characterized by the strata (basal, spiny, granular, and lucid-corneal), with well-evidenced keratinization. The dermis showed intense cellularity (fibroblasts), uniform distribution of collagen fiber bundles, mild inflammatory infiltrate, extensive vascularization, and absence of dermal attachments. The skin fragments observed 3 days after treatment showed the presence of cellular debris, absence of re-epithelialization, moderate cellularity (fibroblasts), intense inflammatory infiltrate with polymorphonuclear predominance in the dermis and associated with subcutaneous adipose tissue, and wide vascularization (Figure 3).

### 2.2. Topical Treatment with Cinnamaldehyde Reduces the Severity of Infection in Animals with Wounds Contaminated by S. aureus

**Figure 4:**
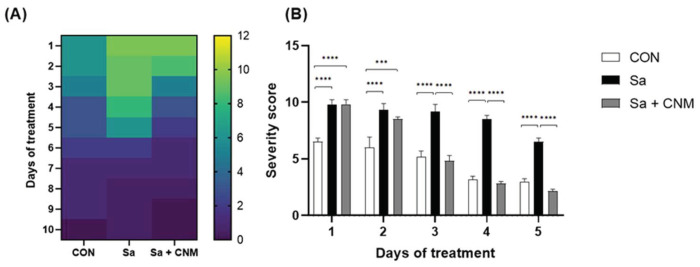
Effects of topical treatment with cinnamaldehyde in the clinical presentation of skin lesion contaminated by *Staphylococcus aureus.* (**A**) Heat map of Severity score during 10 days of treatment. (**B**) Data of severity score for selected days of treatment. CON: animals with uninfected skin lesions; Sa: animals with skin lesions infected with *S. aureus*; Sa + CNM: animals with skin lesions infected with *S. aureus*. *** *p* < 0.001; **** *p* < 0.0001.

During the experiment, macroscopic aspects were analyzed daily to assess the severity of the inflammatory process induced by the skin lesion. Higher severity scores were found for the Sa group between the 1st and 5th days of treatment when compared to the CON group, proving that the presence of *S. aureus* triggers a prolongation of the inflammatory phase (Figure 4). CNM-treated lesions had lower score values than the Sa group, the differences were significant between the 3rd and 5th days (*p* < 0.001). The score values for Sa + CNM and CON groups were similar between the 3rd and 5th days (*p* > 0.05).

### 2.3. Topical Treatment with Cinnamaldehyde Reduces the Levels of Pro-Inflammatory Cytokines in Wounds Contaminated by S. aureus

**Figure 5:**
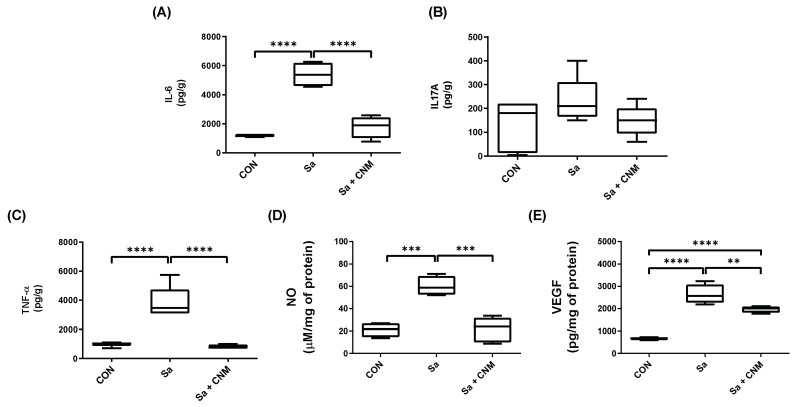
Effects of three days of topical treatment with Cinnamaldehyde on inflammatory mediators present in wound tissue contaminated by *Staphylococcus aureus*. (**A**) IL-6; (**B**) IL-17; (**C**) TNF-α; (**D**) NO; (**E**) VEGF. CON: animals with uninfected skin lesions; Sa: animals with skin lesions infected with *S. aureus*; Sa + CNM: animals with skin lesions infected with *S. aureus*. ** *p* < 0.01; *** *p* < 0.001; **** *p* < 0.0001.

After three days of treatment, IL-6, TNF-α, and IL-17A were detected (Figure 4), while the others evaluated cytokines (IL-2, IL-4, IL-10, and IFN-γ) were not detected. The Sa group presented higher levels of IL-6 (5375.50 ± 728.78 pg/g), TNF-α (3470.33 ± 764.13 pg/g), NO (58.60 ± 6.89 μM/mg of protein), and VEGF (2564.99 ± 296.77 pg/mg of protein) when compared to CON group (1146.90 ± 80.71 pg/g, 1035.54 ± 103.50 pg/g, 21.83 ± 5.41 μM/mg of protein and 684.36 ± 44.41 pg/mg of protein for IL-6, TNF-α, NO, and VEGF, respectively) (Figure 5).

Treatment with CNM promoted significant reductions in tissue concentrations of IL-6 (1889.30 ± 504.90 pg/g, *p* < 0.0001), TNF-α (760.77 ± 120.15 pg/g; *p* < 0.0001), NO (24.08 ± 9.16 μM/mg of protein; p< 0.001), and VEGF (2002.24 ± 99.29 pg/mg of protein; *p* < 0.01), when compared with the Sa group. The inhibition values induced by CNM treatment were in the order of 64.85%, 78.08%, 58.92%, and 21.94% for IL-6, TNF-α, NO, and VEGF (Figure 5), respectively. IL-17 levels were not significantly altered among the experimental groups (Figure 5B). Likewise, on the tenth day, the cytokines were not significantly detected.

### 2.4. Topical Treatment with Cinnamaldehyde Reduces the Neutrophil Levels in Wounds Contaminated by S. aureus

**Figure 6:**
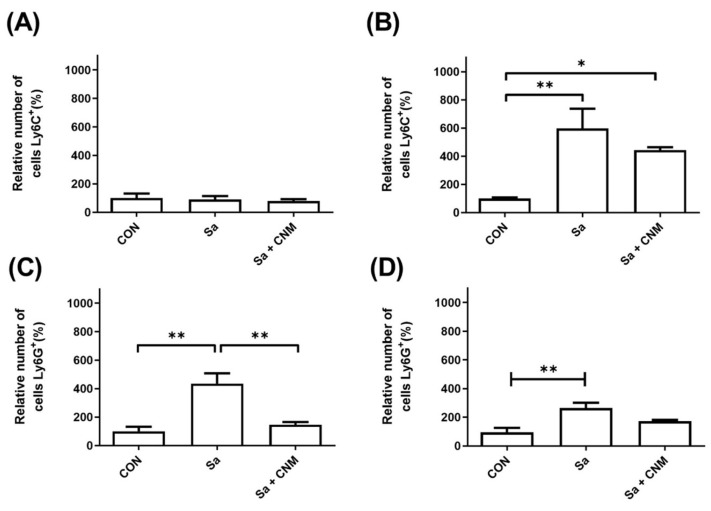
Effects of topical treatment with cinnamaldehyde on the number of macrophages (Ly6C+) and neutrophils (Ly6G+) in the skin and blood of animals submitted to lesions contaminated by *Staphylococcus aureus*. (**A**) Relative amount of Ly6C+ cells in the skin of the different experimental groups; (**B**) Relative amount of Ly6C+ cells in the blood of the different experimental groups; (**C**) Relative amount of Ly6G+ cells in the skin of the different experimental groups; (**D**) Relative amount of Ly6G+ cells in the blood of the different experimental groups. CON: animals with uninfected skin lesions; Sa: animals with skin lesions infected with *S. aureus*; Sa + CNM: animals with skin lesions infected with *S. aureus*. * *p* < 0.05; ** *p* < 0.01.

The populations of monocytes/macrophages (Ly6C+) and neutrophils (Ly6G+) were analyzed in wound samples and the peripheral blood of mice from the different experimental groups (Figure 6). Regarding the levels of macrophages in the skin tissue, it was not possible to observe significant changes among the experimental groups (Figure 6A). On the other hand, the levels of its precursors in the blood (monocytes) were increased (6.7-fold) in the group of animals with lesions infected by *S. aureus* (*p* < 0.001). Importantly, the treatment with CNM reduced the levels of monocytes (about 35%) compared to the Sa + CNM group (Figure 6B). 

Regarding the neutrophils in the wound tissue (Figure 6C), their population was dramatically altered upon cutaneous infection by *S. aureus* (an increase of 335%; *p* < 0.01). This picture was altered in the group submitted to topical treatment with CNM, which had similar neutrophil levels to the group without infection, representing a 2.98-fold reduction in relation to the Sa group (*p* < 0.01). Infection of wounds with *S. aureus* also altered the number of blood neutrophils, an increase of 2.81-fold in relation to the CON group (*p* < 0.01). In the Sa + CNM group, reduced levels of neutrophils were detected in the blood compared to animals with infected wounds (approximately 50%), but also without statistical differences (Figure 6D).

### 2.5. Topical Treatment with Cinnamaldehyde Reduces the Bacterial Load in Wounds Contaminated by S. aureus

**Figure 7:**
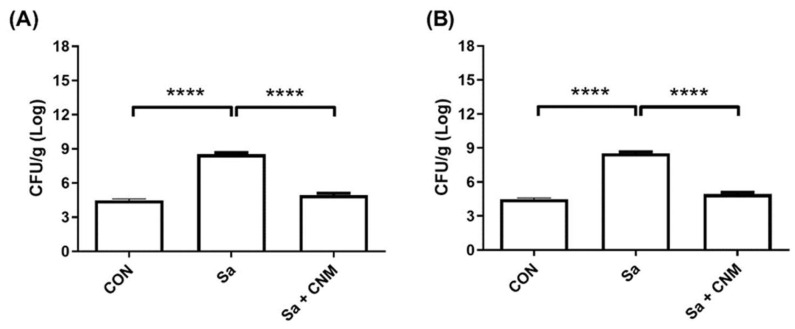
Effects of topical treatment with cinnamaldehyde on bacterial load in wound tissue contaminated by *Staphylococcus aureus* after 3 (**A**) and 10 (**B**) days of treatment. CON: animals with uninfected skin lesions; Sa: animals with skin lesions infected with *S. aureus*; Sa + CNM: animals with skin lesions infected with *S. aureus*. **** *p* < 0.0001.

Finally, it was evaluated whether the treatment with CNM could reduce the *S. aureus* load in the infected wound. On the 3rd day, the Sa group presented an average of 10.29 ± 0.28 Log CFU/g, while Sa + CNM showed 7.36 ± 0.20 Log CFU/g (*p* < 0.0001) (Figure 7A). This pattern was also found after 10 days of treatment where wounds from the Sa group showed 8.49 ± 0.26 Log CFU/g while those from the Sa + CNM group were 4.83 ± 0.23 Log CFU/g (*p* < 0.0001). At this point, the values for the bacterial load of Sa + CNM and CON groups were similar (*p* < 0.05) (Figure 7B). 

## 3. Discussion

The negative effects of infections on the healing of cutaneous wounds denote the importance of the prospection of essential oils and their components as new alternatives for the treatment of these lesions [7,8,17,18]. This work evaluated the effects of CNM in a model of wound infection provoked by *S. aureus*. The main results obtained in this work suggest that the topical treatment of wounds with CNM improves the healing process of cutaneous lesions infected by *S. aureus*, an effect associated with decreased bacterial load and modulation of the host response.

The beneficial effects of topical treatment with CNM on the healing process were reported using experimental wounds in mice, including lesions infected by *P. aeruginosa* [10,19]. The healing action was related to angiogenesis induction at the wound site through the activation of the PI3K/AKT (phosphatidylinositol 3-kinase (PI3K/AKT) and MAPK (mitogen-activated protein kinase) signaling pathways [19]. In wounds infected by *P. aeruginosa*, CNM reduced the expression of inflammatory mediators and microbial proliferation. These effects were mediated by the TRPVA1 receptor [10]. It is noteworthy to highlight that CNM is considered safe at concentrations of up to 8% in human skin tissue and up to 15% in other mammals [20], values higher than the dose used in this present study (0.4%).

The data obtained in this research demonstrate that this compound reduces the exacerbated inflammation related to *S. aureus* infection by decreasing the migration of neutrophils to the lesion site and decreasing the release of pro-inflammatory cytokines (IL-6 and TNF -α). These results are compatible with the anti-inflammatory properties of CNM demonstrated using in vitro and in vivo models of infection [9,10,21,22].

Regulation of the lesion’s inflammatory phase is essential for the tissue repair process. The prolongation of this phase is related to the progression to chronic wounds, due to the destruction of adjacent tissues, which usually leads to a predisposition to systemic infections [3,6,23]. The presence of pathogenic microorganisms and/or necrotic tissues will extend the period of inflammation, causing significant delays in the healing process [5,16,24]. The higher levels of neutrophils, NO, IL-6, and TNF-α in lesions contaminated by *S. aureus* demonstrate this deleterious role of infection during the inflammatory phase.

IL-6 and TNF-α are cytokines that, together with other chemotactic molecules, promote the migration of inflammatory cells and stromal cells to the wound site [25]. Neutrophils are the first to migrate to the site of infection and are activated by microbial structures and/or molecules derived from dead cells. They play crucial roles in the antimicrobial defense and repair process. However, in situations of severe injury, these cells can contribute to an exacerbated inflammatory state that is harmful to the host [26].

Several in vitro reports indicate that CNM interferes with the viability, adhesive properties (including biofilm formation), and toxicity (hemolysin production) of *S. aureus* [13,14,27]. These virulence factors, especially bacterial biofilms, are related to the chronicity of skin lesions [24,28]. However, the in vivo anti-infective efficacy of this compound towards infections caused by *S. aureus* has only been evaluated using larvae of *Galleria mellonella*, an invertebrate organism [14]. The effects of CNM on the growth and expression of virulence factors of *S. aureus* may lead to a reduction in tissue damage and inflammatory process.

## 4. Materials and Methods

### 4.1. Phytocompound

Cinnamaldehyde was obtained from Sigma-Aldrich^®^ (Darmstadt, Germany, purity > 95%).

### 4.2. Animals and Ethical Conditions

This study was approved by the CEUMA University Animal Ethics Committee (Protocol No. 129/17). The *Swiss* mice (~25 g; 6 to 8 weeks) were provided by the CEUMA University animal facility. The thirty-six healthy animals were allocated in individual polypropylene cages arranged in a ventilated rack with independent insufflation and exhaust systems. The experimental model was conducted in an airy room (average temperature of 21 °C) with a 12 h light-dark cycle. During all experimental periods, the animals received water and food *ad libitum*. 

### 4.3. Wound Induction and Topical Treatment 

The dose of CNM used was determined based on its minimum bactericidal concentration (MBC) against *S. aureus* strains (1000 µg/mL) [14]. The topical treatment was based on the administration of 50 µL of a 4000 µg/mL CNM solution (four times MBC), resulting in the application of 200 µg per animal each day. Each mouse was anesthetized by intramuscular injection of xylazine hydrochloride (1 mg/kg) and ketamine chloride (50 mg/kg). The thoracic dorsal region was trichotomized and cleaned before the induction of an excisional wound with blunt-tipped scissors (0.64 cm^2^) and dissecting forceps. Following this, each lesion was infected with 80 μL of a suspension of *S. aureus* ATCC 6538 (1.5 × 10^8^ CFU/mL; based on 0.5 Mcfarland standard), except for a group of animals (CON, *n = 12*) which received 80 μL of 150 mM NaCl (saline solution). 

### 4.4. Clinical Evaluation of the Wounds

One day post-infection, the animals with infected wounds were divided into two groups: mice (*n = 12*) treated daily with a single dose of cinnamaldehyde (200 µg/animal; Sa + CNM) and those that receive sterile 2% DMSO (dimethyl sulfoxide) solution (Sa group, *n = 12*). The CON group was also treated with saline solution. After the procedure, commercial covers (Labor care, Cure aid) were applied in the wounded area. The topical treatment and macroscopic evaluations were conducted within a laminar flow, to minimize the risk of contamination by other microorganisms.

During the experiment, several macroscopic parameters were daily evaluated for the determination of an index of severity by the sum of the values obtained for each parameter: wound area (0–7), amount of exudate (0–3), type of exudate (0–4), edema intensity (0–3), the color of surrounding skin tissue (0–4), and type of debridement tissue (0–3) [10]. The wound area was registered with the caliper and calculated by the equation: π × R × r. “R” and r” are the largest and smallest radiuses, respectively.

Six animals from each group were euthanized by anesthetic overdose on the 3rd and 10th days of treatment and samples of wounded tissue were taken for microbiological, immunological, and histological analyzes, as described in the next section.

### 4.5. Histological Assessment of Tissue Repair

After euthanasia, the skin fragments were collected and fixed in 10% buffered formaldehyde (pH 7.2), followed by dehydration in increasing concentrations of ethyl alcohol, diaphanization in xylene, imbibition, and paraffin embedding. The paraffin blocks obtained were submitted to microtome cuts from 3 µm to 5 µm in thickness, with subsequent staining of the slide containing tissue samples in Hematoxylin and Eosin (HE). Lesions were analyzed by light microscopy, approximately 10 fields, at 40–400× magnifications. The criteria evaluated were cellular debris (presence or absence), inflammatory infiltrate, re-epithelialization (presence or absence), vascularization, and distribution pattern of collagen fibers/fibroblasts. The following scores were established for the inflammatory infiltrate: absence (not observed in any field), mild (1 to 3 observed fields), moderate (4 to 6 observed fields), and intense (above 7 observed fields).

### 4.6. Dosage of Inflammatory Mediators

Tissue samples were processed as described above and used for the determination of cytokine levels (Interleukin-2 (IL-2), Interleukin-4 (IL-4), Interleukin-6 (IL-6), Interferon-γ (IFN-γ), Tumor Necrosis Factor (TNF), Interleukin -17A (IL-17A) and Interleukin-10 (IL-10)). The quantification was performed using the BD Cytometric Bead Array (CBA) Mouse Th1/Th2/Th17 Cytokine Kit (BD Biosciences, Sao Paulo, Brazil) following the manufacturer’s instructions. Data collection was performed in a BD Accuri C6 flow cytometer and analyzed in CBA FCAP Array software (BD Biosciences, Sao Paulo, Brazil). Values were expressed in pg/g.

The levels of Vascular Endothelial Growth Factor (VEGF) in wound tissue were measured by the Mouse VEGF ELISA Kit (Sigma-Aldrich; São Paulo, Brazil), while the amount of nitric oxide (NO) was determined using Griess Reagent [29]. The protein concentration in each sample was quantified by Bradford reagent (Sigma-Aldrich; São Paulo, Brazil) using a standard curve (0–500 µg/mL of bovine serum albumin). The absorbance values were measured at a MB-580 microplate reader (Heales, Shenzhen, China). The levels of NO and VEGF were expressed per mg of total protein.

### 4.7. Characterization of Cell Phenotype by Flow Cytometry

The skin samples were macerated in RPMI medium and filtered through a Corning^®^ cell strainer (Sigma) with 40 µm pores. After filtration, samples were washed twice with phosphate buffer saline (PBS) and resuspended in supplemented RPMI medium (10% fetal serum and penicillin-streptomycin). Approximately one million cells were added to each well of a microdilution plate and labeled with 7AAD (7-aminoactinomycin D; Thermo Fisher Scientific) for viability, and with different monoclonal antibodies (Ly6C, CD19, Ly6G, CD3, CD11b, CD4, CD62L, CD14, and CD3) conjugated to fluorochromes (FITC, PE, PerCP, and APC), all from eBiosciences (São Paulo, Brazil). Samples were analyzed on a BD Accuri C6 flow cytometer (BD Biosciences—Immunocytometry Systems, San Jose, CA, USA). Events were analyzed using FlowJo 7.6.1 software (TreeStar-Ashland, OR, USA).

### 4.8. Quantification of S. aureus Load in the Wound Tissue 

Tissue samples (100 mg) were macerated in 1 mL of PBS with homogenization for 90 s, followed by centrifugation for 5 min at 350× *g*. The supernatants were diluted in PBS and aliquots (5 µL) of each 10-fold dilution were plated in Mannitol salt agar. After incubation at 37 °C for 24 h, the number of bacteria in the tissue was determined and expressed as CFU/g.

### 4.9. Statistical Analysis 

The statistical evaluation of the results was performed in Graphpad Prism 8.4.3. The data were submitted to an analysis of variance (ANOVA), followed by the Bonferroni test. The results were expressed as mean ± standard error. Differences between the experimental groups were considered to be significant when *p* < 0.05.

## 5. Conclusions

Taken together, the data from this study demonstrated that CNM promoted the acceleration of the healing process of wounds contaminated by *S. aureus.* CNM, due to its anti-inflammatory character, modulated the expression of pro-inflammatory mediators leading to lower recruitment of cells to the infected wound. These effects were observed using the inflammation severity score and the dosage of inflammatory mediators. Additionally, treatment with CNM also tended to decrease the relative number of neutrophils and monocytes in the blood. Topical treatment also with CNM reduced the bacterial load in the injured tissue, confirming the previously reported anti-*S. aureus* action. These results corroborate the therapeutic suitability of cinnamaldehyde for wound treatment.

## Data Availability

Data is available upon request.
